# Qualitative Research of Composite Graphene Membranes Using the Electric Mode in SEM and AFM

**DOI:** 10.3390/ma18010163

**Published:** 2025-01-03

**Authors:** Grzegorz Romaniak, Konrad Dybowski, Łukasz Kołodziejczyk, Paulina Kowalczyk

**Affiliations:** Faculty of Mechanical Engineering, Institute of Materials Science and Engineering, Lodz University of Technology, 1/15 Stefanowskiego St., 90-924 Lodz, Poland

**Keywords:** graphene, membrane, filtration, AFM imaging, SEM imaging

## Abstract

The development of new graphene-based materials necessitates the application of suitable material imaging techniques, especially for the identification of defects in the graphene structure and its continuity. For this purpose, it is natural to use one of the main properties of graphene—electrical conductivity. In this work, we prepare a 9 cm^2^ large-area monolayer graphene membrane on porous scaffolding sealed with either GO or rGO. Then, we use electrostatic force microscopy (EFM) AFM mode along with SE and AEE SEM modes to characterize the as-prepared graphene membranes thoroughly. The combination of SEM-AEE and AFM-EFM techniques not only assesses the quality of graphene itself but also characterizes the selectivity and effectiveness of masking graphene layer defects by applying GO or rGO. This makes these methods valuable in optimizing the production of advanced graphene nanocomposites such as semipermeable membranes.

## 1. Introduction

Two-dimensional-structured nanomaterials, such as graphene, hold great potential for the development of filtration membranes, including the separation of ion/molecule contaminants from aqueous solutions [1,2,3]. This potential arises from their unique structural characteristics and excellent physicochemical properties [4,5,6]. Graphene exhibits exceptional mechanical, chemical, electrical, and thermal properties [7,8,9,10,11]. Monolayer graphene is particularly important in the construction of filtration membranes, serving as a single-atom barrier to contaminant migration. Such a thin membrane can potentially significantly reduce the resistance to water molecule flow. Large-area monolayer graphene, used in membrane production, is synthesized via Chemical Vapour Deposition (CVD) on solid or liquid copper [12,13,14,15,16,17,18,19,20] and metallurgical methods on liquid copper (HSMG^®^) [21,22]. This graphene, used for water filtration membranes, cannot have a perfect structure; it must have appropriately sized structural defects that can trap ion/molecule contaminants while allowing water molecules to pass through [23,24,25]. In addition to sub-nanometer structural pores in graphene (ranging from 0.2 to 0.7 nm) capable of separating ions from water, there are larger defects that may be introduced during the graphene synthesis process, transfer procedure, or membrane preparation. Such defects should be detected and characterized, and then effectively eliminated by covering these areas with another carbon material, such as graphene derivatives—graphene oxide (GO); reduced graphene oxide (rGO); or a polymer [26,27,28,29]. Graphene oxide (GO) is primarily produced using the Hummers method. This method results in small graphene flakes with numerous oxygen-containing functional groups (hydroxyl, epoxy, carbonyl, and carboxyl) attached to their edges and surfaces. It can be reduced again through thermal, electrical, or chemical treatment [30,31,32,33] to produce reduced graphene oxide (rGO), which has a structure of small graphene-like flakes with trace amounts of oxygen groups. Depending on the reduction method used, the degree of reduction, hydrophobicity, number of oxygen groups, and electrical properties of rGO can vary, affecting its surface properties, including its ability to adsorb contaminants. Large-area graphene, GO, and rGO are used in the production of new filtration membranes. Here, large-area graphene serves as the filtering material, while GO/rGO is used to mask defects in the graphene layer. The effectiveness of selective defect masking in graphene by GO/rGO depends on the structural characterization methods used for these materials [27,29].

Graphene is primarily characterized by Raman spectroscopy (to determine the number of layers and degree of defects) [34], transmission electron microscopy (structural vacancies, dislocations, and grain boundaries) [35], and scanning tunnelling microscopy which allows for the determination of structural defects and electrical properties [36].

Light microscopy, scanning electron microscopy, and AFM allow for the assessment of microscopic defects on adequate substrates [37,38]. The continuity of the graphene layer after transfer to a polymer substrate can be evaluated using scanning electron microscopy (SEM). A technique that has found particular application in the study of conductive layers, such as graphene on non-conductive substrates (polymers, ceramics), utilizes the signal of electrons absorbed by the sample (AEE mode). The image contrast generated during the measurement results from differences in the currents flowing through the sample depending on whether the electron beam is in a conductive or non-conductive area. Dark areas on a bright (i.e., conductive) background illustrate structural discontinuities in the graphene on a polymer or ceramic substrate.

So far, AFM imaging has not been widely used for membrane characterization purposes. One of the advanced techniques employed for this purpose may be electrostatic force microscopy (EFM), a variant of atomic force microscopy (AFM).

EFM allows for the measurement of the electrostatic force between a probe tip and the sample surface. This is achieved by applying a voltage to the probe tip and measuring the resulting force. The data obtained from EFM can provide valuable information about the surface potential and charge distribution of the sample, which are critical parameters for understanding the behaviour of graphene-based membranes. In the context of water filtration, EFM can be used to investigate the distribution of nanoscale pores in the graphene sheet, which are responsible for the selective permeability of the membrane. By understanding how these pores are distributed and how they interact with water molecules and contaminants, it is possible to optimize the membrane design for maximum filtration efficiency.

In this paper, we present a method for the characterization of nanometric and micrometric defects in graphene using the electrical conductivity of graphene as the main property used for detection. Graphene filtration membranes were fabricated by transferring large-area graphene onto a non-conductive, porous polymer substrate. Then, selective coverage of graphene defects by GO or rGO was performed. Using scanning electron microscopy, with particular emphasis on the AEE mode and one of the atomic force microscope electrical modes, electrostatic force microscopy (EFM), the effectiveness of graphene defect masking was determined. It has been shown that both modes can be used complementary to effectively identify graphene defects and, consequently, serve as useful tools for characterizing new graphene-based semipermeable membranes.

## 2. Materials and Methods

### 2.1. Materials

High-Strength Metallurgical Graphene was synthesized according to the patent (method of producing graphene from liquid metal, US 9284640, USA) and the procedure described in the previous article [39] with minor modifications. As a matrix for graphene synthesis, a 0.2 mm nickel foil electroplated with copper (0.15 mm) in a saturated solution of copper sulphate pentahydrate (CuSO_4_·5H_2_O) was used. Then, Cu/Ni substrates were placed in a SuperCarb Seco Warwick SA generator (Świebodzin, Poland) and heated up to 1060 °C at a pressure of 10 Pa and carburized in an acetylene (0.4 ln/min), ethylene (0.4 ln/min), and hydrogen (0.2 ln/min) mixture. Four cycles each consisting of gas dosing (5 s) and soaking (15 min) were used. Next, the samples were heated up to 1100 °C in a pure argon atmosphere (5 ln/min) and soaked for 5 min to melt Cu. The pressure during the graphene growth stage was 2 kPa. Finally, the samples were cooled down with the reactor to room temperature.

After synthesis graphene was characterized via Raman spectroscopy and was confirmed to be a monolayer with limited defects [39,40]. The graphene nanostructure was characterized by a TEM Talos F200X from FEI (Hillsboro, OR, USA). The previous studies identified and described nanostructural defects with sizes ranging from 0.2 to 0.5 nm [22,39].

MicroPES^®^ 1F EL (3M, Jüchen, Germany) porous support substrate with a thickness of 110 µm, a pore diameter of 0.1 to 1.0 μm, and a pore density 1.05 × 10^6^ mm^−2^ was used for graphene transfer.

Poly(methyl methacrylate) (PMMA) with a molecular weight of 996,000 g/mol from the Aldrich Chemistry Company (St. Louis, MO, USA), dissolved in chlorobenzene (Chempur, Piekary Śląskie, Poland) at a concentration of 0.10 mol dm^−3^ (9.2 g of PMMA per 100 mL of chlorobenzene) was used to cover the graphene during the transfer procedure.

The solvent used to dissolve and remove the PMMA film from the graphene during the transfer procedure was 2-propanol of technical grade 70% (Chempur, Poland).

Graphene oxide with 0.4% dispersion in water (4 mg mL^−1^) was purchased from the Advanced Graphene Products Company, Zielona Góra, Poland.

For the reduction of graphene oxide, diluted commercial 80% hydrazine hydrate from POCh, Gliwice, Poland was used.

### 2.2. Graphene Transfer

The HSMG^®^ graphene transfer process onto polysulfone membrane was conducted using a modified wet transfer method, in which the graphene growth substrate etching was replaced by graphene electrochemical delamination [40]. Firstly, graphene samples on the Cu/Ni substrate were cut, straightened, and then drop/blade coated with 10 µL cm^−2^ PMMA solution. Then, the polymer was dried at 50 °C for 40 min. The graphene electrochemical delamination from the growth substrate was conducted on a laboratory semi-automatic stand according to the parameters described elsewhere [40,41]. Then, graphene on PMMA was washed twice with deionized water. After that, it was placed on a water-wetted polysulfone membrane, rolled, and dried at 70 °C for 5 min. To remove the PMMA, the as-prepared stack was placed in a boiling isopropanol vapour for one hour and then washed twice in cold isopropanol.

### 2.3. GO and rGO Application

Reduced graphene oxide was prepared using a 1% solution of hydrazine in 10 mL of a 0.25% GO suspension in DI water and left for 1 h to reduce at room temperature (RT). The solution was sonicated before the addition of the hydrazine. The properties of GO and rGO were described in a previous work [41].

Graphene membranes were drop-coated with 200 µL of GO or rGO (both 0.25% suspension in DI water), respectively. The suspensions were applied on the membrane surface, and after 3 min, the excess was poured off. The suspensions were sonicated for 1 min before being applied. The as-prepared membranes were baked at 80 °C for 30 min.

### 2.4. Optical Microscopy and SEM Examination

Graphene membrane analysis was conducted on a Nikon Eclipse MA200 light microscope (Nishioi, Japan) with inverted optics. Then, the same areas were identified and analyzed using the HITACHI S 3000 N scanning electron microscope (Tokyo, Japan). The tests were performed at an accelerating voltage of 5 kV, using SE (secondary electrons) and AEE (absorbed electron emission) detectors. The AEE imaging mode is better suited for this purpose, as it shows the contrast of the image resulting from the differences in the conductivity of the tested surface areas. The combination of the two images from the SE and AEE modes provides an effective assessment of the continuity of the graphene layer, even in areas where the substrate pores are present.

### 2.5. AFM Examination

Surface morphology, topography, and local electrical properties measurements were performed under ambient conditions using a Multimode 5 atomic force microscope equipped with a Nanoscope V controller (Bruker Corporation, Billerica, MA, USA) operating in one of its electrical modes—EFM. Commercial silicon cantilevers of type HQ: NSCPt15 (MicroMasch, Wetzlar, Germany) with a platinum-coated tip radius of <30 nm, a cantilever spring constant of 40 N/m, and a resonant frequency of 325 kHz were used. Image acquisition was performed using Nanoscope 7.3 software, and further image processing was carried out using Nanoscope Analysis 1.9 (Bruker Corporation). For the AFM/EFM imaging, the membrane substrates were attached to a magnetic puck using electrically conductive Cu tape along with the use of Ag conductive paste to connect the top of the membrane (with graphene structures) to the puck surface.

The EFM method allows primarily for the qualitative mapping of surface potential. Electrostatic force microscopy measurements were made in a two-pass (lift) mode and the size of the images was up to 100 × 100 μm. The electrostatic force was measured as a change in the cantilever resonant frequency (phase shift) due to the differences in charge between the sample and the tip. The lift height and DC bias were set to 100 nm and −4 V, respectively.

## 3. Results and Discussion

*Optical Microscopy Characterization*

**Figure 1:**
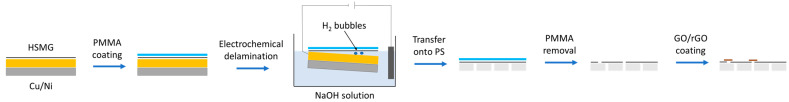
Graphene membrane preparation scheme.

Graphene membranes prepared according to Figure 1 were proven to be semipermeable in our previous studies [39,41]. The key components in these membranes are active monolayer graphene with subnanometric pores and a sealing material (GO or rGO). Using rGO as a sealing material enhances both solute rejection and water flow. Previous studies conducted for HSMG^®^ graphene showed that the use of only a monolayer of quaimonocrystalline metallurgical graphene does not result in obtaining a semipermeable membrane, mainly due to the presence of transfer defects and defects from the synthesis process. Optimizing the graphene transfer method and effectively sealing these defects enables an increase in osmotic water flow (25 mL h^−1^ m^−2^ bar^−1^) and rejection rate (up to 95%). Understanding the characteristics of graphene defects and the effectiveness of sealing are important steps in membrane preparation methodology. The use of electrical modes based on the electrical conductivity of the characterized material offers a natural approach to identifying graphene on a non-conductive substrate.

**Figure 2:**
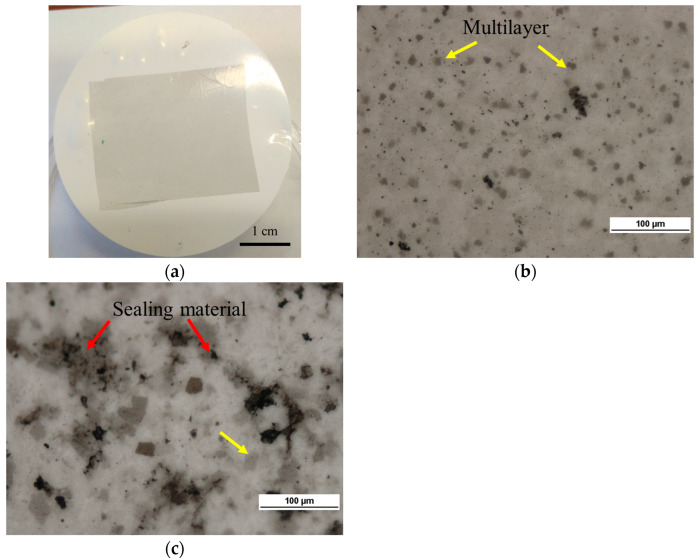
Graphene membranes. (**a**)—bare graphene on a porous substrate. (**b**)—an optical image of graphene on a porous substrate. (**c**)—rGO selectively sealed graphene membrane.

The membranes on PS porous substrate with a graphene layer size of 9 cm^2^ were fabricated (Figure 2a). Then, GO or rGO was applied as a defect-sealing material. The microscopic graphene quality was evaluated. The bare graphene layer on the porous substrate (Figure 2b) is mostly monolayer (bright grey area) with multilayers visible as darker, flat areas. Sealing material on graphene was observed under an optical microscope as a black, uneven layer (Figure 2c).

**Figure 3:**
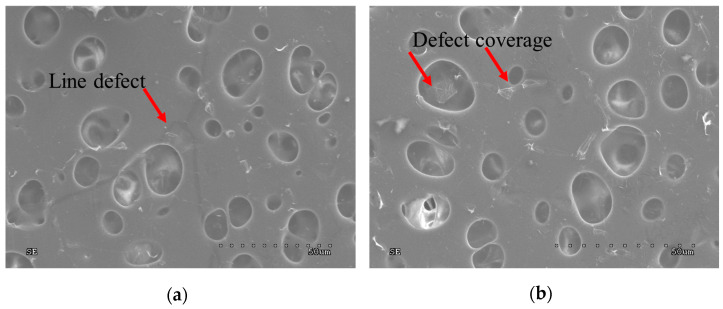
(**a**)—graphene with line defects on a porous substrate. (**b**)—selectively sealed graphene membrane.

Figure 3a shows the graphene layer transferred onto PS with line defects in the graphene (marked with a red arrow). Despite these defects, graphene is stretched over the pores of the supporting substrate. After sealing with rGO (Figure 3b) platelets of rGO are arranged along a line, in this case, on a shape similar to a hexagon (the discontinuity occurring at the border of graphene crystallites has been covered). The size of the hexagons is of the order of tens of micrometres, which corresponds to the size of the hexagons in graphene after synthesis. rGO was also deposited in the pore region of the substrate, which is a crucial area to be sealed when it comes to filtration.

**Figure 4:**
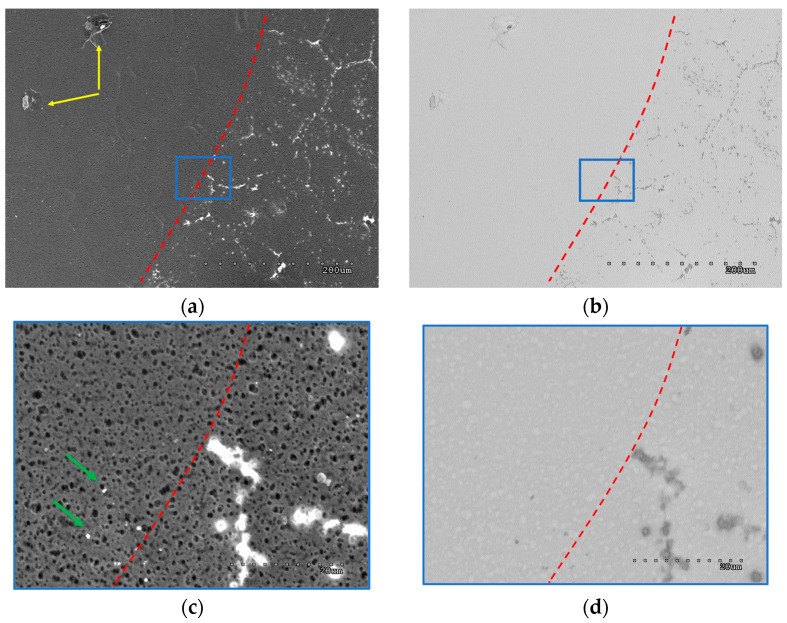
(**a**–**d**) graphene membrane sealed with rGO. The left side of the images is covered with sealing material, and the right side is bare graphene on a porous substrate. SEM images, SE mode (**a**–**c**), and AEE mode (**b**,**d**). (**c**,**d**) are the enlarged areas marked with a blue frame.

Figure 4a–d presents the boundary of rGO sealing applied on the graphene membrane (boundary marked with the red dotted line, rGO applied on the left side of the images). Most of the defects are in substrate unevenness that reflects copper grains (HSMG^®^ graphene growth substrate). This may be due to the presence of defects in the graphene itself from the synthesis or a privileged place for the formation of transfer defects. Both SE and AEE modes in SEM show good masking of these discontinuities. Almost all point discontinuities were covered. A large part of the continuous, defect-free graphene has been preserved uncoated. Yellow arrows indicate rGO conglomerates, which are not common due to the sonication of rGO solution before application on the membrane. Effective sonication also has an impact on obtaining a thin masking layer, wherein the former grain boundary, despite the linear defect being sealed, and substrate pores are still visible in the SEM image (Figure 4c). This sealing method is not 100% efficient. In Figure 4c, there are still graphene clustered point defects that have not been sealed (bright areas, marked with green arrows).

Next, optical microscopy, SEM, and AFM analysis were conducted in selected areas with larger graphene discontinuities to better determine the possibility of imaging different membrane areas. The imaging was carried out in such a way that all the membrane components were present in each analyzed area. On the images from the optical microscope, for ease of use, the areas are marked according to Table 1.

**Figure 5:**
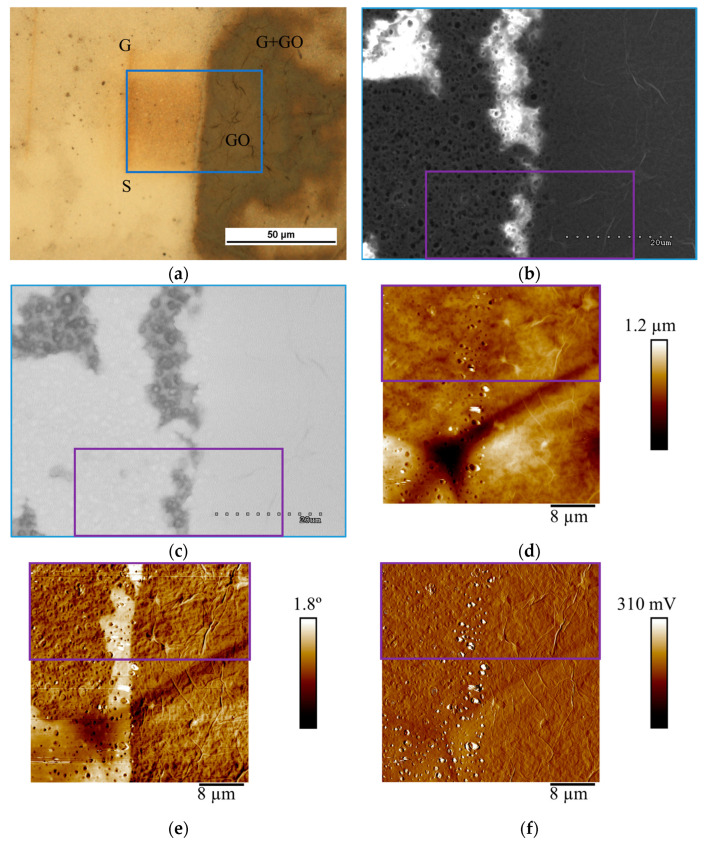
Imaging the effectiveness of covering graphene discontinuities by selectively applied GO. (**a**)—optical microscopy. (**b**)—SEM SE and (**c**)—SEM AEE images of the composite membrane. (**d**–**f**)—atomic force microscopy images in topography, EFM, and amplitude modes, respectively.

Figure 5 shows the selective coverage of graphene defects by graphene oxide. SEM images in SE and AEE modes (Figure 5b,c) allow us to observe both large areas of graphene discontinuity (right area in Figure 5b) and identify places completely covered by GO. On the left side is a part completely covered with GO, while in the upper left corner, there is a selectively deposited GO layer on graphene. In this area, substrate pores are visible in areas covered only with graphene. The defects in graphene have almost completely been masked by GO. Here, too, no substrate pores are visible. In SE mode, the graphene oxide morphology is visible (characteristic wrinkles). In AEE mode (Figure 5c), the image of the surface coverage with graphene and graphene oxide is similar to Figure 5b. Imaging in the conduction mode gives a contrasting image of the surface—the areas covered with graphene or graphene oxide are bright, while the areas not covered with graphene are grey. In the areas not covered with graphene, pores of the polysulfone substrate can be seen. However, there is no difference in imaging the areas covered with graphene and those covered with graphene oxide, although graphene oxide conducts electricity much worse. Then, atomic force microscopy imaging was performed for the area marked in purple. GO wrinkles are visible in AFM imaging in all three modes. The electric mode (EFM) imaging (Figure 5e) allowed us to obtain a contrast between the layer covered by large-area graphene and the bare substrate (bright areas). The boundary of graphene coverage and open pores are visible. Open pores are also visible in the amplitude method, but in this case, it is impossible to distinguish the graphene coverage boundary in the area between the opened pores. Thus, the EFM mode, due to the electrical properties of both large-area graphene and GO, turned out to be effective in characterizing the performance quality of composite graphene membranes.

**Figure 6:**
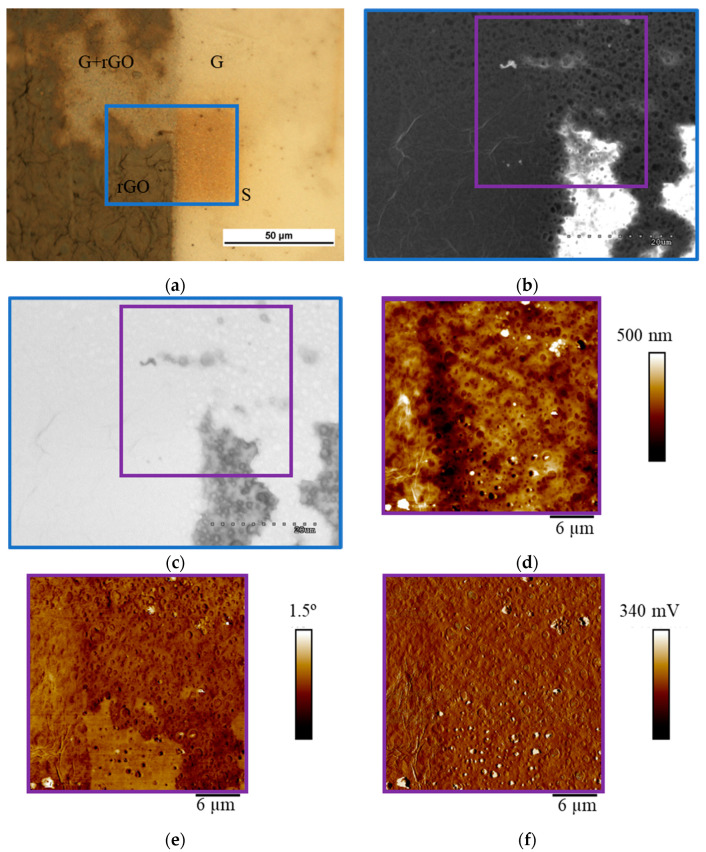
Imaging the effectiveness of covering graphene discontinuities by selectively applied rGO. (**a**)—optical microscopy. (**b**)—SEM SE and (**c**)—SEM AEE images of the composite membrane. (**d**–**f**)—atomic force microscopy images in topography, EFM, and amplitude modes, respectively.

Imaging of sealing graphene defects with rGO (Figure 6) was conducted the same way as GO sealing (Figure 5). High-quality graphene in the upper left corner, despite being exposed to the rGO aqueous suspension, was not covered with rGO platelets, thus the pores of the substrate under graphene are still visible in SEM images (Figure 6b). This phenomenon was confirmed on EFM imaging (Figure 6e) where large-area graphene is not covered by rGO. This is related to the hydrophobic properties of graphene. The rGO aqueous suspension only covers the places where the graphene layer is damaged, because the porous polysulfone substrate exhibits hydrophilic properties.

The images from the SEM microscope—SE mode (Figure 6b) and SEM—AEE mode (Figure 6c) compared with the images obtained from the AFM microscope show the full correlation of the obtained test results. The same areas not covered with graphene can be seen. In Figure 6b, the pores of the substrate can be seen in both graphene-coated and non-graphene-coated areas. Only the contrast of the image can indicate the lack of coverage in this place—the areas not covered with graphene are bright. In contrast, no substrate pores are visible in the rGO-coated areas due to the thickness of the rGO coating. The results obtained in Figure 6c can be interpreted similarly. The AEE mode indicates good conductivity of the substrate (bright areas) in the graphene and reduced graphene oxide-coated areas. Non-conductive areas where graphene or rGO is absent are grey. In these images, the substrate’s pores are visible only in the non-graphene-coated areas. It is also difficult to distinguish which area is covered only with graphene and which with reduced graphene oxide. No characteristic wrinkles of the coating can be seen here for rGO. The results obtained in AEE mode are in full agreement with those obtained in SE mode. Analyzing the images captured with the AFM microscope (Figure 6d–f), it can be concluded that the results obtained in the topography mode do not allow for an accurate interpretation of the quality of the graphene coating on the membrane, and for an assessment of the effectiveness of the sealing of this layer of rGO. Visible here are the pores of the substrate, basically regardless of the area of the membrane, and point-like particles, probably small rGO conglomerates or graphene impurities. In the lower corner, the finely outlined wrinkle shape of the rGO coating can be observed. EFM mode (Figure 6e) gives a more complete result. The substrate’s pores (dark dots) are clearly visible in areas not covered with graphene. The rGO-coated sites do not have visible pores, while the graphene-coated pores are also imaged, but in a different way than on bare polysulfone. Here, one can clearly distinguish between pores covered by graphene (they look like convex particles) and pores over which there is no stretched graphene (dark dots). In the amplitude mode (Figure 6f), porosity is also visible both on the substrate not covered with graphene and where it is present. Here, it is also possible to indicate pores covered by a graphene layer and those where graphene is damaged. As before, the areas where rGO is present are difficult to pinpoint unambiguously. These are areas where the pores are not well mapped. Similarly to the case of coating graphene membranes with graphene oxide, the EFM mode proved to be equally effective in imaging areas covered with graphene and sealed with rGO.

## 4. Conclusions

The characterization of graphene defects and the evaluation of the effectiveness of their sealing are important steps in the methodology of preparing graphene membranes for water filtration. The selectivity and efficiency of such membranes ultimately depend on the accuracy and precision of these studies. The use of electrical modes based on the electrical conductivity of the characterized material is a natural approach to identifying graphene on a non-conductive substrate such as polysulfone. As demonstrated in this article, the use of such test methods as the SEM–AEE mode and AFM–EFM mode provides the opportunity for good characterization of the continuity of graphene layers and the effectiveness of their sealing with GO and rGO. Interestingly, although the difference in the conductivity of GO and rGO is significant, it did not fundamentally affect the imaging efficiency of the methods mentioned above. However, it should be noted that the use of only one test method may produce inconclusive results. The combination of SEM-AEE and AFM-EFM techniques yields more reliable results. Both areas are not covered by a graphene layer and the absence of graphene on individual pores of the polysulfone substrate can be seen. These techniques also make it possible to assess the selectivity and effectiveness of masking graphene layer defects by applying GO or rGO. In summary, imaging techniques based on differences in the electrical conductivity of the tested composite materials are highly effective for characterizing the quality of graphene filtration membranes.

## Figures and Tables

**Table 1:** Signatures used to describe components of the graphene membrane on figures.

Signature on Figures	Membrane’s Area
S	Bare substrate
G	Graphene layer
GO	Graphene oxide on PS
rGO	Reduced graphene oxide on PS
G + GO	Graphene oxide selectively coating graphene defects
G + rGO	Reduced graphene oxide selectively coating graphene defects

## Data Availability

The original contributions presented in the study are included in the article, further inquiries can be directed to the corresponding author.
